# Awareness, attitudes, and utilization of large language models among healthcare students in Saudi Arabia: a cross-sectional analysis

**DOI:** 10.3389/fmed.2026.1935478

**Published:** 2026-09-16

**Authors:** Mohammed A. Almeshari, Osama A. Asiri, Ayedh D. Alahmari, Abdulrhman S. Alghamdi, Sulaiman S. Alsaif, Saad A. Alhammad, Yasser K. Bamogaddam, Malak Alowaisi, Ali M. Alasmari, Nowaf Y. Alobaidi, Faraj K. Alenezi, Khalid S. Alwadeai

**Affiliations:** 1 Department of Rehabilitation Health Sciences, College of Applied Medical Sciences, King Saud University, Riyadh, Saudi Arabia; 2 Institute of Inflammation and Ageing, University of Birmingham, Birmingham, United Kingdom; 3 Respiratory Therapy Department, College of Medical Rehabilitation Sciences, Taibah University, Madinah, Saudi Arabia; 4 Department of Respiratory Therapy, College of Applied Medical Sciences, King Saud Bin Abdulaziz University for Health Sciences, Alahsa, Saudi Arabia; 5 King Abdullah International Medical Research Center, Alahsa, Saudi Arabia; 6 Ministry of the National Guard Health Affairs, Alahsa, Saudi Arabia; 7 College of Applied Medical Sciences, King Saud Bin Abdulaziz University for Health Sciences, Alahsa, Saudi Arabia; 8 King Abdullah International Medical Research Center, Riyadh, Saudi Arabia; 9 Department of Anesthesia, Ministry of the National Guard Health Affairs, Riyadh, Saudi Arabia

**Keywords:** artificial intelligence, ChatGPT, health professions education, healthcare students, large language models, medical education, Saudi Arabia

## Abstract

**Introduction:**

Large language models (LLMs) such as ChatGPT are increasingly adopted in health professions education worldwide, yet evidence on their use among healthcare students in the Middle East remains limited.

**Methods:**

This cross-sectional study assessed awareness, attitudes, and utilization of LLMs among healthcare students across Saudi Arabian universities using a self-administered online questionnaire, adapted from a previously published instrument, distributed to healthcare students at multiple Saudi universities between April and August 2025. Descriptive and inferential statistics were used to compare responses by sex. Of 449 responses collected, 441 provided informed consent; after applying eligibility criteria, 435 were included in the final analysis (70.3% female; median age 21.0 years).

**Results:**

Most students (78%) reported familiarity with LLMs and 87% agreed they are useful for both students and educators, though 75% acknowledged the risk of inaccurate information and only 18% had attended formal LLM training. Female students reported significantly higher perceived usefulness of LLMs than males (*p* = 0.011), while males were more likely to report low understanding of LLM functionality (*p* = 0.009).

**Discussion:**

These findings reveal a substantial gap between LLM adoption and structured AI-literacy training among healthcare students in Saudi Arabia, suggesting a need for curricula that build critical appraisal and verification skills alongside safe LLM use.

## 1 Introduction

Medical education has undergone significant technological evolution over the past several decades, transitioning from traditional didactic approaches to interactive, technology-enhanced learning modalities (1, 2). The integration of educational technology in healthcare training has progressed through multiple stages—from computer-assisted platforms to virtual reality simulations, mobile learning applications, and, most recently, artificial intelligence (AI)-based educational tools (3, 4). This evolution reflects the growing recognition that modern healthcare professionals require not only comprehensive medical knowledge but also proficiency in utilizing the advanced technological tools that are increasingly prevalent in clinical practice (5).

The rapid advancement of AI technologies, particularly large language models (LLMs), is fundamentally reshaping the delivery of medical education. As healthcare systems embrace digital transformation and data-driven decision-making, the integration of AI-powered educational tools has emerged as a critical factor in preparing future healthcare professionals for increasingly complex clinical environments. This technological shift is particularly significant for emerging healthcare markets such as Saudi Arabia, where substantial investments in healthcare infrastructure and education align with the Kingdom's Vision 2030 strategic initiatives (6).

LLMs, exemplified by systems such as ChatGPT (OpenAI, San Francisco, California), Claude (Anthropic, San Francisco, California), and Gemini (Google DeepMind, Mountain View, California), represent a new paradigm in educational technology with the capacity to transform how medical knowledge is acquired, processed, and applied (7, 8). These advanced AI systems, trained on vast corpora of medical literature and clinical data, can generate human-like text responses, deliver personalized learning experiences, and support complex clinical reasoning tasks (9). Their potential applications in medical education span multiple domains, including curriculum development, personalized learning pathways, virtual patient simulations, clinical decision support, and formative assessment (7, 10).

Saudi Arabia's medical education system has experienced remarkable growth, expanding from no medical schools in the 1960s to a total of 37 medical colleges, spanning both government and private sectors, today (11). This expansion reflects the Kingdom's commitment to developing a robust healthcare workforce capable of meeting the evolving health needs of its population (6).

### 1.1 Theoretical context

The adoption and utilization of LLMs in medical education can be interpreted through the lens of the Technology Acceptance Model [TAM; Davis, 1989 (12)], which posits that perceived usefulness and perceived ease of use are the primary determinants of technology adoption behavior. We draw on TAM here as an interpretive conceptual perspective for contextualizing the attitudinal and behavioral patterns observed in this study, rather than as a structural model formally tested through validated TAM constructs or path analysis; our cross-sectional survey did not measure perceived usefulness and perceived ease of use as latent psychometric variables. Within this interpretive frame, TAM helps explain why some students readily integrate LLMs into their study routines while others remain hesitant (13). Recent empirical work applying TAM-informed frameworks to LLM adoption among medical students similarly found that perceived usefulness is a strong predictor of behavioral intention to use LLMs, an effect mediated by attitudes toward the technology, while perceived risk and social influence also shape adoption decisions (14). Additional theoretical insight is drawn from cognitive load theory [CLT; Sweller, 1988 (15)], which we likewise apply as a complementary interpretive lens rather than a tested model. CLT suggests that LLMs may reduce extraneous cognitive load by providing readily accessible explanations and summaries, potentially freeing cognitive resources for deeper learning. However, this same mechanism raises concerns about “cognitive offloading”—the risk that students may delegate essential cognitive work to AI rather than engaging in the effortful processing necessary for durable learning and clinical reasoning development (16, 17).

### 1.2 Current evidence on LLM use in health professions education

In this study, “health professions” encompasses the broad range of clinical training programs represented in our sample, including medicine, dentistry, pharmacy, nursing, and allied health and rehabilitation disciplines such as respiratory therapy, physical and occupational therapy, speech-language pathology and audiology, clinical laboratory sciences, radiology and imaging technology, anesthesia technology, emergency medical services, clinical nutrition, and public health—each with distinct curricular structures, clinical training requirements, and licensure pathways that may shape how students engage with LLMs.

Studies examining student perceptions of AI in educational settings reveal generally positive attitudes, with healthcare students showing particular enthusiasm for AI tools that enhance learning efficiency and provide access to current medical information (9, 18). A systematic review of health students’ knowledge and perceptions of AI reported significant variations in attitudes and literacy across disciplines and demographics, with senior students and those pursuing research careers demonstrating more favorable outlooks (19). A multinational cross-sectional survey of 391 medical students from more than 30 countries found that while awareness of AI was high (91.6%), only 34.5% believed their institutions were prepared for AI integration, and 82.1% expressed interest in structured AI training (20). Similarly, a large-scale international survey of medical stakeholders found an overall generative AI awareness rate of 93.3%, with moderate knowledge and generally positive attitudes (21).

Beyond general-purpose information seeking, LLMs are increasingly applied to specific, high-stakes academic tasks in health professions training. Emerging work has explored their use in preparing for practical and licensing assessments, including simulating standardized patients, generating case scenarios, and supporting self-assessment for Objective Structured Clinical Examinations (OSCEs) (22). Such discipline- and task-specific applications are directly relevant to the survey items in the present study assessing whether students use LLMs to clarify medical topics and supplement textbook learning, and underscore the need for evidence on how these emerging use cases intersect with assessment integrity and competency development.

Despite the substantial potential benefits, several challenges must be addressed for successful LLM implementation. A primary concern is the phenomenon of “hallucination,” whereby LLMs generate responses that appear plausible but contain factual inaccuracies or fabricated information (23). A recent Viewpoint in *The Lancet Digital Health* highlighted that while AI holds considerable promise for transforming medical training through high-fidelity clinical simulations and personalized learning, significant ethical concerns remain regarding the unclear boundaries of appropriate AI use, calling for clear guidelines to govern its integration (24). In medical education, where precision is paramount, such errors carry adverse implications for student learning and subsequent clinical practice (25). A systematic review of LLMs in medical education identified incorrect responses, overreliance on technology, erosion of critical thinking, and academic integrity concerns as the most frequently cited limitations (25). Furthermore, a randomized controlled trial demonstrated that standard warning disclaimers about ChatGPT's fallibility did not significantly alter medical students’ diagnostic decision-making behavior, suggesting that simple cautionary measures may be insufficient to calibrate trust in AI-generated advice (26).

There is also the risk that overreliance on AI may diminish students’ critical thinking skills and capacity for independent clinical judgment (16, 17). A systematic review of ChatGPT applications in dental education reported that a substantial proportion of included studies identified the risk of biased or incorrect responses, potential clinical errors, and concerns about loss of critical thinking (27).

### 1.3 Study rationale and objectives

While the global evidence base on LLMs in medical education is growing rapidly, the majority of studies originate from Western educational contexts. Limited data exist regarding LLM utilization patterns, training gaps, and attitudinal nuances among healthcare students in the Middle East, where cultural, linguistic, and curricular factors may influence technology adoption differently (20, 21). This study aimed to provide a comprehensive assessment of how healthcare students in Saudi Arabia utilize LLMs in their education, examining awareness, attitudes, utilization patterns, and perceived impact on learning outcomes. We sought to characterize the current landscape of LLM utilization in Saudi medical education and identify critical training needs that can inform curriculum development.

## 2 Materials and methods

### 2.1 Study design and setting

This study employed a cross-sectional design utilizing an online, self-administered survey hosted on Google Forms. The survey was distributed electronically to healthcare students enrolled at Saudi universities across various regions of the country through institutional email lists, official university communication networks, and social media platforms, as detailed in Section 2.4. Data collection was conducted between April 2025 and August 2025 (approximately five months). Ethical approval was obtained from the King Saud University Institutional Review Board (IRB No. E-25-9618; approval date: 8 April 2025), prior to the start of data collection. This study is reported in accordance with the Checklist for Reporting Results of Internet E-Surveys (CHERRIES) and the Strengthening the Reporting of Observational Studies in Epidemiology (STROBE) guidelines for cross-sectional studies.

### 2.2 Target population and eligibility

The target population consisted of students enrolled in healthcare-related degree programs at Saudi universities, spanning pre-professional (foundation-year), undergraduate clinical, internship, and graduate/postgraduate academic levels. Participants represented 17 predefined health science majors captured by the survey instrument—Medicine, Dentistry, Pharmacy, Nursing, Respiratory Therapy, Physical Therapy, Occupational Therapy, Speech-Language Pathology, Audiology, Clinical Laboratory Sciences, Radiology and Imaging Technology, Anesthesia Technology, Emergency Medical Services, Clinical Nutrition, Biomedical Technology, Public Health, and Health Promotion—plus an open-text “Other” option that captured additional allied health programs (e.g., optometry, midwifery, dental technology, health information management). Academic level was categorized as pre-medical (foundation or common first year), Year 2 through Year 5 or above (discipline-specific clinical training years, whose length varies across programs—six years in medicine and dentistry, five years in pharmacy and optometry, and four years in other allied health disciplines—before all students proceed to internship), internship (the mandatory one-year post-graduate clinical training required of all healthcare disciplines represented in this study), and graduate/postgraduate students pursuing advanced degrees. Inclusion criteria were: (1) current enrollment in a healthcare-related program at a Saudi university, and (2) willingness to participate voluntarily. Eligibility was assessed via self-reported major, academic level, and university location within Saudi Arabia, consistent with the approved IRB protocol; practicing healthcare professionals no longer enrolled as students were excluded.

### 2.3 Sample size and sampling

A total of 449 responses were collected. Of these, 441 (98.2%) provided electronic informed consent; the remaining 8 declined consent and were not included in any subsequent processing or analysis. Among the 441 consenting respondents, 6 were subsequently excluded for ineligible profession (i.e., the respondent's self-reported program of study did not correspond to a healthcare-related field), yielding a final analytic sample of 435 participants.

According to the Ministry of Education's 2021–2023 Higher Education Report, 40,255 students were enrolled in healthcare-related programs across Saudi Arabia. Using a 95% confidence level, a 5% margin of error, and a conservative response proportion of *p* = 0.50 (which maximizes the required sample size), the minimum sample size was calculated to be 381 students using the Cochran formula for proportions in cross-sectional surveys (28). A convenience sampling approach was adopted to maximize participation, supported by the wide availability of institutional and social media communication channels. Participants were enrolled at both public (92.2%) and private (7.8%) Saudi universities across all five administrative regions of the country (Central, Western, Eastern, Northern, and Southern; Table 1), and represented the 17 predefined health science majors plus additional allied health programs specified via free text (Section 2.2). The survey did not capture individual institution names, only university type and geographic region; consequently, we are unable to report the exact number of participating universities, and this is noted as a limitation (Section 4.7). Given the convenience sampling strategy and reliance on institutional and social media recruitment channels, findings should be interpreted as representative of the surveyed sample of healthcare students rather than as nationally representative of all Saudi healthcare students.

**Table 1:** Demographic characteristics of study participants stratified by sex (*N* = 435).

Characteristic	Overall (*N* = 435)	Female (*n* = 306)	Male (*n* = 129)	*p*-valueᵃ
Age, median (IQR)	21 (20, 22)	21 (20, 22)	22 (20, 23)	<.001
Academic year				<.001
Pre-medical	42 (9.7)	30 (9.8)	12 (9.3)	
Year 2	83 (19.1)	74 (24.2)	9 (7.0)	
Year 3	118 (27.1)	76 (24.8)	42 (32.6)	
Year 4	78 (17.9)	45 (14.7)	33 (25.6)	
Year 5+	19 (4.4)	10 (3.3)	9 (7.0)	
Internship	44 (10.1)	33 (10.8)	11 (8.5)	
Graduate/postgraduate	51 (11.7)	38 (12.4)	13 (10.1)	
University type				.005*
Public	401 (92.2)	275 (89.9)	126 (97.7)	
Private	34 (7.8)	31 (10.1)	3 (2.3)	
Region				<.001
Central	239 (54.9)	184 (60.1)	55 (42.6)	
Western	107 (24.6)	60 (19.6)	47 (36.4)	
Eastern	41 (9.4)	32 (10.5)	9 (7.0)	
Southern	23 (5.3)	12 (3.9)	11 (8.5)	
Northern	25 (5.7)	18 (5.9)	7 (5.4)	
Weekly study hours				.028
< 5 h	102 (23.4)	63 (20.6)	39 (30.2)	
5–10 h	210 (48.3)	154 (50.3)	56 (43.4)	
10–20 h	83 (19.1)	65 (21.2)	18 (14.0)	
> 20 h	40 (9.2)	24 (7.8)	16 (12.4)	
Computer literacy				.073
Beginner	99 (22.8)	65 (21.2)	34 (26.4)	
Intermediate	292 (67.1)	215 (70.3)	77 (59.7)	
Advanced	44 (10.1)	26 (8.5)	18 (14.0)	
Attended LLM workshop	79 (18.2)	49 (16.0)	30 (23.3)	.077*

Values are *n* (%) unless otherwise indicated. IQR, interquartile range; LLM, large language model. ᵃWilcoxon rank-sum test for age; Pearson's chi-square or Fisher's exact test for categorical variables.

University type and workshop-attendance *p*-values were recalculated using Fisher's exact test (previously.006 and.068 respectively, computed via Pearson's chi-square without confirming expected-cell-count assumptions); the exact number of participating universities was not captured by the survey instrument and could not be reported (Section 2.3).

*p < 0.05, denoting a statistically significant difference between sexes.

### 2.4 Recruitment procedures

Participants were recruited through multiple channels, including institutional email lists, official university networks, and social media platforms (Twitter [now X], WhatsApp, and Telegram groups). Recruitment messages contained study details, eligibility criteria, and an electronic link to the survey hosted on Google Forms (Google LLC, Mountain View, California). Eligibility was not independently verified against institutional enrollment records; ineligible respondents were expected to self-exclude based on the eligibility criteria and consent information presented at the start of the survey, and ineligible submissions were subsequently identified *post hoc* from self-reported major, academic level, and university location within Saudi Arabia, consistent with the approved IRB protocol (Section 2.3). Google Forms was configured to restrict submissions to one response per Google account (verified via the respondent's linked email address) to minimize duplicate entries; this account information was used solely for duplicate prevention and was not linked to survey responses in the analytic dataset, preserving participant anonymity.

### 2.5 Data collection and instrument

Data were collected using a structured, self-administered 31-item questionnaire (Supplementary Data Sheet S1). The instrument comprised one electronic informed-consent item; 12 items capturing demographic and study-habit characteristics (age, sex, nationality, academic year, major/program, university type, geographic region, weekly study hours, GPA, computer literacy, frequency of computer/tablet use for medical study, and prior LLM workshop attendance); and 18 five-point Likert-scale items (1 = strongly disagree to 5 = strongly agree) assessing three domains—Knowledge/awareness of LLMs (6 items), Attitudes toward LLMs in healthcare education (6 items), and Practice/utilization of LLMs (6 items). The questionnaire was adapted from a previously published instrument originally developed and validated by Biri et al. (2023) among undergraduate medical students, made available under a Creative Commons Attribution (CC-BY) license permitting academic use, distribution, and adaptation with appropriate citation (29). We refer to it here as an adapted, rather than independently validated, instrument for the present population: no additional forward–backward translation, expert content review, or pilot testing was conducted prior to administration, as the source instrument had previously demonstrated adequate psychometric performance in a comparable population. This is noted as a limitation (Section 4.7).

Responses were recorded directly in Google Forms and stored in a secure, password-protected electronic database. All responses were anonymized to protect participant identity. Only the research team had access to the dataset. Participation was voluntary, and informed consent was obtained electronically prior to survey completion. Data cleaning and validation were performed prior to analysis, including screening for implausible values (e.g., a small number of internally inconsistent age entries, which were treated as missing for age-specific descriptive statistics only; Section 3.1).

### 2.6 Statistical analysis

Data were analyzed using R (30) within the RStudio integrated development environment (version 2024.09.1; Posit Software, PBC, Boston, Massachusetts). Descriptive statistics (frequencies and percentages for categorical variables; medians and interquartile ranges for continuous variables) were used to summarize demographic characteristics and responses to all 30 substantive survey items (excluding the consent item). Sex (male vs. female) was the primary comparison variable examined across three groups of variables: (1) demographic and study-habit characteristics (age, academic year, university type, region, weekly study hours, computer literacy, and workshop attendance; Table 1); (2) the six Knowledge/awareness items; and (3) the twelve combined Attitude and Practice items (Table 2). For the continuous variable age, the Wilcoxon rank-sum test was used to compare sexes. For categorical variables, Pearson's chi-square test was used when all expected cell counts were ≥5; Fisher's exact test (or, for 5 × 2 Likert-item tables, a Monte Carlo-simulated exact test based on 20,000 replications) was used otherwise, which applied to several Likert-item comparisons and to 2 × 2 comparisons (e.g., university type, workshop attendance) with small expected counts. Given the number of sex comparisons performed across survey items, results for individual items should be interpreted as exploratory and hypothesis-generating; we did not apply a formal multiple-comparison correction, and we acknowledge an elevated risk of Type I error when interpreting any single item-level *p*-value in isolation. A two-tailed significance level of *p* < 0.05 was used for all analyses.

**Table 2:** Distribution of responses to LLM awareness, attitude, and utilization items (*N* = 435).

Survey item	Strongly disagree n (%)	Disagree n (%)	Neutral n (%)	Agree n (%)	Strongly agree n (%)	*p*-value (sex)
*Awareness*						
Familiar with LLMs	11 (2.5)	9 (2.1)	77 (17.7)	170 (39.1)	168 (38.6)	.098
Understand how LLMs generate responses	20 (4.6)	56 (12.9)	124 (28.5)	156 (35.9)	79 (18.2)	.009*
Aware LLMs may give wrong information	8 (1.8)	13 (3.0)	85 (19.5)	175 (40.2)	154 (35.4)	.013*
Useful for both teachers and students	8 (1.8)	7 (1.6)	40 (9.2)	141 (32.4)	239 (54.9)	.017*
Help simplify complex concepts	6 (1.4)	15 (3.4)	60 (13.8)	168 (38.6)	186 (42.8)	.139
Complement traditional learning materials	4 (0.9)	7 (1.6)	50 (11.5)	172 (39.5)	202 (46.4)	.107
*Attitudes*						
Open to LLMs as extra learning tools	8 (1.8)	16 (3.7)	68 (15.6)	169 (38.9)	174 (40.0)	.953
Provide comprehensive medical information	8 (1.8)	16 (3.7)	90 (20.7)	190 (43.7)	131 (30.1)	.037*
Colleges should promote LLM use	8 (1.8)	6 (1.4)	99 (22.8)	183 (42.1)	139 (32.0)	.091
Could reshape medical learning	5 (1.1)	9 (2.1)	76 (17.5)	182 (41.8)	163 (37.5)	.391
Overreliance may harm clinical reasoning	12 (2.8)	46 (10.6)	95 (21.8)	159 (36.6)	123 (28.3)	.322
Risk of learning wrong information	6 (1.4)	18 (4.1)	96 (22.1)	173 (39.8)	142 (32.6)	.806
*Utilization*						
Use LLMs to clarify medical topics	7 (1.6)	19 (4.4)	52 (12.0)	181 (41.6)	176 (40.5)	.409
Found new resources via LLMs	11 (2.5)	36 (8.3)	93 (21.4)	167 (38.4)	128 (29.4)	.391
Use LLMs if books lack the information	18 (4.1)	96 (22.1)	94 (21.6)	132 (30.3)	95 (21.8)	.731
Use LLMs if Google doesn't help	29 (6.7)	110 (25.3)	87 (20.0)	126 (29.0)	83 (19.1)	.306
Adapt self-study methods using LLMs	15 (3.4)	83 (19.1)	144 (33.1)	140 (32.2)	53 (12.2)	.746
More confident discussing topics with LLMs	15 (3.4)	31 (7.1)	135 (31.0)	157 (36.1)	97 (22.3)	.057

LLM, large language model. All items were measured on a 5-point Likert scale (1, strongly disagree; 5, strongly agree).

The *p*-value column (sex comparison) is newly added in this revision: Pearson's chi-square test on the full 5 × 2 response table, or a Monte Carlo-simulated exact test (20,000 replications) where expected cell counts were <5 (marked *), consistent with the updated Statistical Analysis section (2.6). Full sex-stratified *n* (%) for all categories and items are provided in Supplementary Table S1.

## 3 Results

### 3.1 Participant characteristics

Because program length and curricular structure vary across the represented health science disciplines (e.g., six-year programs in medicine and dentistry, five-year programs in pharmacy and optometry, and four-year programs in other allied health disciplines, each culminating in a mandatory one-year internship), “academic year” reflects each participant's self-reported standing within their own program rather than a uniform metric of training duration across disciplines; the distribution below should be interpreted with this heterogeneity in mind.

To assess whether academic year (as a proxy for educational maturity) was associated with LLM engagement, we compared familiarity, understanding of LLM functionality, use of LLMs for clarifying medical topics, self-study adaptation, and confidence in discussing medical subjects across the seven academic-year groups using the Kruskal–Wallis test, and workshop attendance using the chi-square test. No statistically significant differences emerged for any of these measures (all *p* > 0.11), including understanding of LLM functionality (*p* = 0.315) and workshop attendance, which ranged from 10.3% to 31.0% across groups (*p* = 0.139). Given the small size of some subgroups (e.g., Year 5+, *n* = 19) and the number of comparisons performed, these results should be interpreted as exploratory and hypothesis-generating rather than confirmatory.

Among the 435 participants included in the final analytic sample (recruitment and eligibility flow described in Section 2.3), 306 (70.3%) were female and 129 (29.7%) were male. The vast majority were Saudi nationals (*n* = 429; 98.6%). Four age entries (0.9%) contained internally inconsistent values (e.g., ages inconsistent with reported academic level) and were treated as missing for age-specific descriptive statistics only; all other responses from these participants were retained in the analysis. Based on the remaining 431 valid entries, the median age was 21.0 years (IQR: 20.0–22.0; range: 18–46), with male students being significantly older than female students (*p* < 0.001). All participants met the minimum eligibility age of 18 years.

Students were distributed across different stages of healthcare education: Year 3 (*n* = 118; 27.1%), Year 2 (*n* = 83; 19.1%), Year 4 (*n* = 78; 17.9%), graduate or postgraduate (*n* = 51; 11.7%), internship (*n* = 44; 10.1%), pre-medical (*n* = 42; 9.7%), and Year 5 or above (*n* = 19; 4.4%). The distribution of academic years differed significantly by sex (*p* < 0.001), with a higher proportion of males in Year 3 (33%) and Year 4 (26%) compared to females (25% and 15%, respectively), while Year 2 had a substantially higher representation of female students (24% vs. 7.0%). Most participants were enrolled at public universities (*n* = 401; 92.2%), with a significantly higher proportion of males at public institutions (98% vs. 90%; *p* = 0.005, Fisher's exact test).

Regarding geographic distribution, more than half of the participants studied in the Central region (*n* = 239; 54.9%), followed by the Western (*n* = 107; 24.6%), Eastern (*n* = 41; 9.4%), Northern (*n* = 25; 5.7%), and Southern (*n* = 23; 5.3%) regions. Geographic distribution differed significantly by sex (*p* < 0.001), with females more concentrated in the Central region (60%) and males more represented in the Western region (36%).

Regarding weekly study time, 210 participants (48.3%) reported studying 5–10 h per week, 102 (23.4%) fewer than 5 h, 83 (19.1%) 10–20 h, and 40 (9.2%) more than 20 h, with a significant sex difference observed (*p* = 0.028). Two-thirds of students (*n* = 292; 67.1%) self-identified as intermediate in computer literacy, 99 (22.8%) as beginners, and 44 (10.1%) as advanced, with no significant sex difference (*p* = 0.073). The majority of students (*n* = 295; 67.8%) reported daily use of a computer or tablet for medical studies. The complete demographic characteristics stratified by sex are presented in Table 1.

### 3.2 LLM awareness, understanding, and workshop attendance

Most students reported familiarity with LLMs such as ChatGPT, Google Gemini (formerly known as Google Bard), Microsoft Bing, or Perplexity, with 78% agreeing or strongly agreeing (*p* = 0.098 for the sex comparison). The survey instrument, adapted from an earlier published source (Section 2.5), used the original item wording “Google Bard”; we refer to this tool as Google Gemini (formerly known as Google Bard) elsewhere in the manuscript to reflect its current name. This broad familiarity did not consistently translate into a deeper understanding of how these tools function: only 54% agreed or strongly agreed that they understood how LLMs generate responses, while 29% were neutral and 18% disagreed or strongly disagreed—indicating a gap between recognizing LLMs as tools and understanding their underlying mechanics. A notable sex difference emerged for this item, with male students more likely to strongly disagree with understanding LLM functionality (9.3% vs. 2.6%; *p* = 0.009), despite the absence of a significant sex difference in overall familiarity.

Despite this widespread familiarity, only 79 students (18.2%) reported having attended a workshop or training session on LLMs, with no significant difference between sexes (16% of females vs. 23% of males; *p* = 0.077, Fisher's exact test). Considered together, these findings suggest that students’ engagement with LLMs has developed largely through informal, self-directed exposure rather than structured instruction, which may help explain the gap between high familiarity and comparatively modest functional understanding described above.

### 3.3 Perceptions of LLM accuracy

Building on the awareness patterns described above, perceptions of LLM accuracy were notably cautious: 75% of students acknowledged that LLMs may generate incorrect information (40% agree, 35% strongly agree), even though only 54% reported understanding how these tools generate responses (Section 3.2). This pattern suggests that students’ caution about LLM accuracy does not depend on a sophisticated understanding of underlying model mechanics, and may instead reflect a more general awareness that AI-generated content requires verification. Female students demonstrated significantly higher rates of agreement that LLMs can generate incorrect information compared to males (*p* = 0.013), with 44% of females agreeing versus 30% of males.

### 3.4 Attitudes toward LLMs in healthcare education

The majority of respondents (87%) agreed that LLMs could be useful for both students and educators, with female students endorsing this view at significantly higher rates (60% strongly agreeing vs. 43% of males; *p* = 0.017). Similarly, 82% believed that LLMs help simplify complex medical concepts (39% agree, 43% strongly agree, no significant sex difference), and 86% agreed that LLMs can complement traditional learning materials such as textbooks, notes, and e-books.

Support for promoting LLMs in healthcare education was reported by 74% of respondents (42% agree, 32% strongly agree, no significant sex difference). Overall, 79% believed that LLMs could reshape how students learn and access medical knowledge (no significant sex difference), and 74% agreed that LLMs would help by providing comprehensive medical information, with significantly higher agreement among female students (*p* = 0.037). Concerns about overreliance were common: 65% agreed that excessive reliance on LLMs might impair clinical reasoning skill development, and 72% acknowledged the risk of learning incorrect information and emphasized the need for critical verification; neither of these overreliance-related items showed a significant sex difference.

### 3.5 Utilization patterns

The most prevalent use of LLMs was to obtain clearer explanations of medical topics being studied (82%; 42% agree, 40% strongly agree, no significant sex difference). Additionally, 67% reported that LLMs had introduced them to new study resources and references. Approximately half of participants (52%) used LLMs when textbook information was insufficient, and 48% did so when search engines failed to provide adequate answers; none of these utilization-pattern items showed a statistically significant sex difference.

### 3.6 Impact of LLM use on learning behavior

Distinct from the utilization patterns above, we separately examined whether LLM use was associated with self-reported changes in learning behavior and confidence. Here, 44% of students reported adapting their self-study methods based on insights from LLMs (no significant sex difference), and 58% indicated that LLM use had increased their confidence in discussing medical subjects (36% agree, 22% strongly agree). The complete distribution of responses across all 18 survey items, together with sex-comparison *p*-values, is presented in Table 2 and illustrated graphically in Figure 1; the full sex-stratified response counts and percentages for all 18 items are provided in Supplementary Table S1.

**Figure 1:**
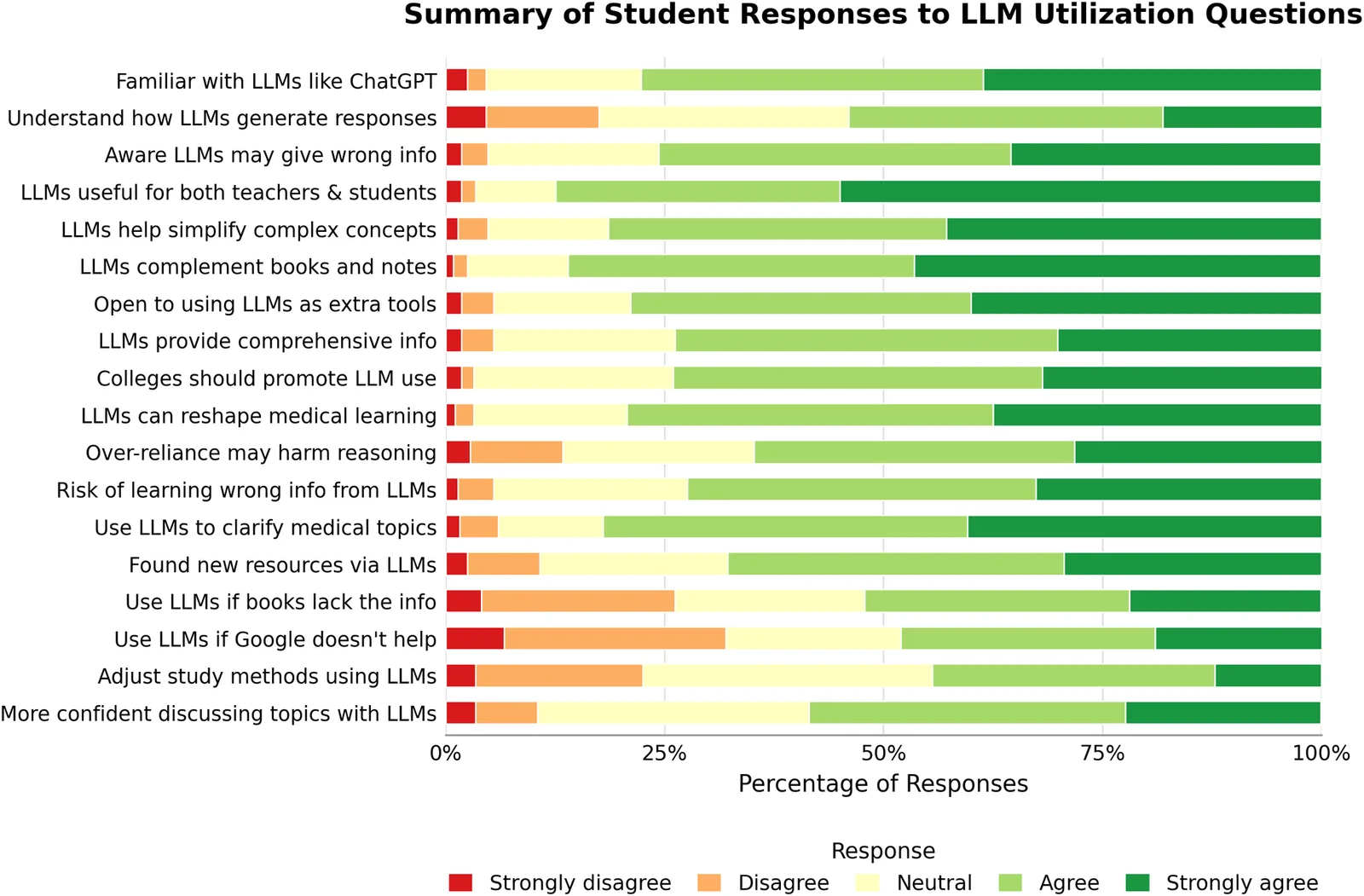
Summary of student responses to LLM utilization survey items (*N* = 435). Horizontal stacked bars represent the distribution of Likert-scale responses (strongly disagree to strongly agree) for each survey item. Items are grouped by domain: awareness (top), attitudes (middle), and utilization patterns (bottom). LLM, large language model.

## 4 Discussion

### 4.1 Principal findings and contextualization

The findings of this study reveal that Saudi healthcare students are actively engaging with LLM technologies, with 78% reporting familiarity with tools such as ChatGPT and 82% using them to clarify medical topics. These findings align with global trends documenting high adoption rates of generative AI among health professions students (7, 9). A recent multinational cross-sectional survey of 391 medical students from more than 30 countries reported an awareness rate of 91.6%, although only 58.1% had access to AI-integrated educational tools, and merely 34.5% believed their institutions were adequately prepared for AI integration (20). Similarly, a study of information-seeking patterns among medical students found that approximately half had used ChatGPT or other LLMs for educational purposes (31). A cross-sectional study among genetic counseling students and faculty found that students primarily used ChatGPT for writing support and concept explanation, with both groups expressing concern about content inaccuracies (32).

While the utilization rate in our sample was high, only 18% of respondents had attended a formal workshop on LLM use. This gap between adoption and training is a notable finding that mirrors the international landscape; however, as discussed further in Section 4.6, our cross-sectional data cannot establish why attendance was low, and this finding should not be read as evidence that additional workshops alone would close the gap. Hack et al. (20) found that 82.1% of students expressed interest in structured AI training, yet institutional preparedness remained low. A national survey of Canadian medical students similarly reported near-universal LLM use, yet found that only a minority felt adequately educated on data privacy regulations applicable to these tools, and a majority agreed that medical schools should integrate formal LLM instruction (33). Consistent with that caveat, we frame structured LLM instruction as an area meriting further investigation rather than a conclusion directly demonstrated by these survey data.

### 4.2 Sex differences in LLM perceptions

An important contribution of this study is the examination of sex differences in LLM attitudes. Female students endorsed the usefulness of LLMs at significantly higher rates than males (*p* = 0.017), with 60% of female students strongly agreeing that LLMs are useful for both teachers and students compared to 43% of males. Female students also demonstrated significantly greater acknowledgment that LLMs can generate incorrect information (*p* = 0.013). Consistent with the moderated interpretation recommended for exploratory, multiple-comparison item-level findings (Section 2.6), we restrict our interpretation to the observed response distributions: in this sample, higher endorsement of usefulness among female students co-occurred with higher awareness of accuracy limitations, without a corresponding sex difference in overreliance concern or utilization frequency (Sections 3.4–3.6). These sex-related differences contrast with several international studies that have reported no significant sex differences in medical students’ attitudes toward LLMs or ChatGPT (34), while others have reported higher AI enthusiasm or usage frequency among male students, suggesting that gender dynamics in AI adoption may vary by population, cultural context, and the specific attitude or behavior measured.

### 4.3 Accuracy awareness and critical appraisal

Participants demonstrated a cautious appraisal of LLM-generated content. Although students utilized LLMs to simplify complex concepts (82%), 75% acknowledged that these models may produce incorrect information—a well-documented phenomenon referred to as hallucination (23). The finding that 72% of students explicitly stated they would not blindly trust LLM-generated information is particularly reassuring and suggests an awareness of the need for critical verification.

This pattern is consistent with recent evidence. A systematic review of LLMs in medical education identified content inaccuracy, overreliance, and erosion of critical thinking as the most frequently reported limitations (25). Furthermore, Kıyak et al. (26) demonstrated in a randomized controlled trial that standard fallibility warnings did not meaningfully alter medical students’ diagnostic behavior when receiving ChatGPT-attributed feedback. The students’ weight-of-advice was significantly lower than the established meta-analytic average, suggesting that perceived LLM credibility was already near a behavioral floor. The authors proposed a “credibility threshold” concept, below which additional cautionary cues have limited effect—a finding that may help explain the cautious appraisal observed in our cohort.

### 4.4 The tension between perceived benefit and overreliance

A notable tension emerged from our data: while 79% of students believed that LLMs could reshape medical learning and 58% reported increased confidence in discussing medical subjects, 65% simultaneously expressed concern that overreliance on these tools may weaken clinical reasoning skills. Similarly, 70%–82% of students reported high day-to-day informational use of LLMs (Sections 3.5–3.6) while also demonstrating high awareness of inaccuracy risk (Section 3.3), indicating that heavy use and critical awareness are not mutually exclusive in this sample. This finding reflects students’ nuanced understanding of AI as both a potential accelerator and a potential threat to their professional development; whether this awareness translates into behaviors that actually mitigate overreliance (e.g., independent verification of LLM output against primary sources) could not be determined from the present cross-sectional, self-reported design and represents a priority for future research combining survey and behavioral or performance-based measures (Section 4.7).

The concern regarding cognitive offloading is well supported by current evidence. Sirag and Moloney (16) cautioned that overreliance on AI tools may inadvertently undermine the experiential learning process essential for developing clinical decision-making competencies. Medical education fundamentally emphasizes the *process* of arriving at clinical decisions, not merely the outcome. When students delegate cognitive effort to an algorithm, they risk attenuating the very reasoning skills essential for safe clinical practice (17). A systematic review of ChatGPT in dental education found that concerns regarding misinformation, overreliance, and reduced critical thinking were frequently reported across included studies (27).

These findings can be interpreted through the lens of cognitive load theory (15). While LLMs may beneficially reduce extraneous cognitive load by providing accessible explanations, they may simultaneously reduce germane cognitive load—the productive effort required to construct durable knowledge schemas. Educators must therefore ensure that the increased confidence reported by students reflects genuine understanding achieved through active engagement rather than superficial familiarity obtained through AI-generated summaries.

### 4.5 Context-specific considerations

This study provides an important Middle Eastern perspective to a field predominantly represented by Western research. The high utilization rates observed in Saudi Arabia reflect, at least in part, the Kingdom's Vision 2030 digital transformation goals and the broader cultural emphasis on technological innovation (6). A recent cross-sectional study among medical students and interns in the western region of Saudi Arabia similarly documented strong student interest in AI applications, with participants expressing enthusiasm for learning about AI's strengths and weaknesses in medicine and the ethical considerations surrounding its use (35). The multinational survey by Luo et al. (21) found that awareness rates and attitudes toward generative AI varied significantly by geographic region and were influenced by education level and work experience, highlighting the importance of context-specific evidence.

An important consideration specific to the Saudi context is the availability of Arabic-language training resources and the performance of LLMs in medical Arabic, given that Saudi healthcare curricula are delivered primarily in English while students’ first language is typically Arabic. This survey did not directly assess whether language (e.g., navigating English-language LLM outputs and interfaces, or comparing LLM performance in Arabic versus English medical terminology) presented a barrier to use; our instrument captured overall familiarity, attitudes, and utilization, but no item specifically addressed linguistic barriers. We are therefore unable to draw direct conclusions about the role of language from our data, and we flag this as a gap for future instruments to address explicitly rather than infer. Ahimaz et al. (32) similarly noted that LLMs have documented limitations with non-English datasets, reinforcing the need for context-appropriate validation of LLM performance in Arabic-language medical education.

### 4.6 Implications for medical education practice

The data demonstrate that LLMs are already embedded in student learning behaviors, rendering prohibition impractical. We offer the following as potential considerations for future curricular development and interventional research, rather than as definitive recommendations that follow directly from this cross-sectional, descriptive survey; their effectiveness would need to be evaluated prospectively.

First, institutions could consider AI literacy workshops or curricular modules that extend beyond basic prompting techniques to encompass verification strategies, source evaluation, and recognition of hallucinated content. We note that only 18% of our sample had attended any such workshop; however, our data cannot distinguish whether this reflects limited institutional workshop availability, low promotion or accessibility of existing offerings, low perceived value among students, or scheduling and competing demands, and we caution against interpreting low attendance as evidence that more workshops alone would close the gap. The systematic review by Shishehgar et al. (19) reported that students consistently identified universities as the optimal setting for learning about AI technologies in healthcare. Any such workshops should explicitly address discipline-specific failure modes, with the goal of helping students develop calibrated trust and supervisory competence over AI-generated outputs.

Second, assessment methodologies could be reconsidered to de-emphasize pure factual recall, which LLMs can readily replicate, in favor of oral examinations, practical simulations, and case-based reasoning exercises in which students articulate and defend their clinical decision-making processes—an approach consistent with competency-based medical education frameworks that value higher-order cognitive skills. We note this as a direction warranting formal evaluation rather than an implication directly demonstrated by our survey data.

Third, structured reflective activities in which students compare LLM-generated responses with evidence from peer-reviewed literature, clinical guidelines, and expert opinion represent a plausible avenue for fostering critical appraisal skills while reinforcing the role of AI as a learning supplement rather than a substitute for clinical reasoning; we present this as a hypothesis for future interventional studies rather than a proven strategy.

### 4.7 Limitations

This study has several limitations. First, the cross-sectional design captures utilization patterns at a single point in time and does not permit tracking how LLM use evolves as students progress through their education. Second, the use of self-reported data introduces the possibility that students may overestimate their understanding of LLM functionality, confuse perceived confidence with demonstrated learning outcomes, or overstate their actual frequency of use relative to general familiarity; future studies could complement self-report with behavioral or performance-based measures. Third, the convenience sampling approach, which relied partly on social media channels, may have introduced selection bias toward technology-savvy students, potentially inflating utilization estimates; we could not calculate a response or non-response rate, as the number of students who viewed but did not complete the survey was not captured by the platform. Fourth, the study did not assess the quality or accuracy of students’ interactions with LLMs, or any objective measure of learning or clinical-reasoning performance, which would require experimental or observational methodologies. Fifth, the predominance of participants from the Central and Western regions, and from public universities, limits the generalizability of findings; we were also unable to report the exact number or discipline-level breakdown of participating universities, as this was not captured by the survey instrument (Section 2.3). Sixth, the questionnaire was adapted from a previously validated instrument without independent forward–backward translation, expert content review, or pilot testing in the current population, which may affect content validity for this specific sample (Section 2.5); our research team plans to conduct formal psychometric re-validation in future work. Seventh, academic-year comparisons should be interpreted cautiously given substantial variation in program length and structure across the represented disciplines; in exploratory testing, we found no statistically significant differences in familiarity, functional understanding, utilization, self-study adaptation, confidence, or workshop attendance across academic-year groups, though several subgroups were small and multiple comparisons were performed, limiting our power to detect true differences (Section 3.1). Eighth, the survey did not directly assess potential linguistic barriers to LLM use in a predominantly Arabic-speaking population studying an English-language curriculum (Section 4.5). Ninth, given the number of exploratory sex comparisons performed across survey items without correction for multiple testing, individual item-level *p*-values carry an elevated risk of Type I error and should be interpreted as hypothesis-generating (Section 2.6). Finally, the sex imbalance (70.3% female) may limit the power to detect some sex differences and may reflect the demographic composition of the accessible student population rather than the overall healthcare student body.

## 5 Conclusion

In this sample, Saudi healthcare students reported using LLMs as a valued, though imperfect, educational tool—applying these technologies to clarify complex medical topics and reporting increased confidence, while also expressing awareness of accuracy limitations. The findings indicate a substantial gap between widespread LLM familiarity (78%) and formal training (18% workshop attendance) in this surveyed population, which we interpret as a potential opportunity, rather than a demonstrated deficiency, for health professions education to address. Female students in our sample reported both higher perceived usefulness and greater awareness of accuracy limitations than male students; given the exploratory, cross-sectional nature of these comparisons, we present this as an observed pattern warranting further study rather than a general claim about sex-based approaches to AI adoption. We suggest that medical education programs and policymakers consider structured AI-literacy curricula as one potential approach to help students use LLMs as a scaffold for critical thinking rather than a substitute for clinical reasoning, with future longitudinal and experimental studies needed to evaluate the impact of such interventions on learning outcomes, clinical reasoning development, and patient safety.

## Data Availability

The raw data supporting the conclusions of this article will be made available by the authors, without undue reservation.

## Supplementary material

Supplementary Data Sheet S1 Full 31-item survey instrument (PDF printout), provided in place of the description “validated questionnaire” per journal request (Section 2.5).

Supplementary Table S1 Full sex-stratified response counts and percentages for all 18 Knowledge/Attitude/Practice Likert items (*N* = 435).
