# Supplementary material for: Awareness, attitudes, and utilization of large language models among healthcare students in Saudi Arabia: a cross-sectional analysis
Source: Front Med (Lausanne). 2026 Sep 16;13:1935478. doi: 10.3389/fmed.2026.1935478 (PMC13623567; doi:10.3389/fmed.2026.1935478)
Supplement: Supplementary file 1 [file Datasheet1.pdf]

Assessing the Utilization of Large Language Models (AI) in Medical Education

This study aims to explore the role of Large Language Models (LLMs) in medical education, examinin their impact on student learning, comprehension, and engagement. By assessing how healthcare students interact with AI-driven tools like ChatGPT, this research seeks to identify the benefits and challenges associated with their use in academic settings. Your participation will provide valuable insights that can help educators and policymakers integrate these technologies effectively into medica curricula.

Participation in this study is completely voluntary, and all responses will be kept strictly confidential. The survey will take approximately 7-10 minutes, and no personally identifiable information will be collected. Your input will contribute to a better understanding of AI's role in medical education and hel shape its future implementation in learning environments. If you agree to participate, please select "I Agree" to proceed with the survey.

This study has been approved by the Institutional Review Board (IRB) at King Saud University (IRB No: E-25-9618).

If you have any questions or concerns about this study, please contact the Principal Investigator, Dr. Mohammed Almeshari, at malmeshari@ksu.edu.sa.

* Indicates required question

1. By selecting **"I Agree"** below, you acknowledge that you have read and understood the information provided, and you voluntarily agree to participate in this study.

   Mark only one oval.

   - I Agree (Proceed to the survey)
   - I Do Not Agree (Exit the survey)

Demographics

2. What is your age? *

   ____

3. What is your gender? *

Mark only one oval.

- Male
- Female

4. What is your nationality? *

Mark only one oval.

- Saudi
- Non-Saudi

5. What year are you currently in? *

Mark only one oval.

- Pre-medical (Foundation/Common First Year)
- Year 2
- Year 3
- Year 4
- Year 5+
- Internship
- Graduate/Postgraduate student

6. Which major or academic program are you enrolled in? *

Mark only one oval.

- Respiratory Therapy
- Medicine
- Dentistry
- Pharmacy
- Anesthesia Technology
- Clinical Laboratory Sciences
- Radiology and Imaging Technology
- Emergency Medical Services (EMS)
- Occupational Therapy
- Physical Therapy
- Clinical Nutrition
- Biomedical Technology
- Public Health
- Health Promotion
- Speech-Language Pathology
- Audiology
- Nursing
- Other:

7. Which type of medical school are you attending? *

Mark only one oval.

- Public
- Private

8. Where in Saudi Arabia is the university you are enrolled in based? *

Mark only one oval.

- Central
- Eastern
- Western
- Southern
- Northern

9. What is your approximate weekly study time outside of class? *

Mark only one oval.

- Less than 5 hours
- 5–10 hours
- 10–20 hours
- More than 20 hours

10. What is you GPA (out of 5)? *

____

11. How would you rate your overall computer literacy? *

Mark only one oval.

- Beginner (Basic use of a computer, web browsing, emails)
- Intermediate (Comfortable with Microsoft Office, basic troubleshooting, software use)
- Advanced (Can use specialized software, troubleshoot problems, basic programming)

12. How often do you use a computer or tablet for medical studies? *

Mark only one oval.

- Daily
- A few times a week
- A few times a month
- Rarely

13. Have you attended any workshops or training sessions about LLMs? *

Mark only one oval.

- Yes
- No

Knowledge of medical students on LLMs in medical education

14. I am familiar with LLMs like ChatGPT, Google Bard, Microsoft Bing, or Perplexity *

Mark only one oval.

- Strongly agree
- Agree
- Neutral
- Disagree
- Strongly disagree

15. I understand how LLMs generate information and responses *

Mark only one oval.

- Strongly agree
- Agree
- Neutral
- Disagree
- Strongly disagree

16. LLMs can generate wrong information *

Mark only one oval.

- Strongly agree
- Agree
- Neutral
- Disagree
- Strongly disagree

17. LLMs can be used both by teachers and students *

Mark only one oval.

- Strongly agree
- Agree
- Neutral
- Disagree
- Strongly disagree

18. Using LLMs helps me simplify complicated medical concepts *

Mark only one oval.

- Strongly agree
- Agree
- Neutral
- Disagree
- Strongly disagree

19. LLMs can help along with traditional learning materials like textbooks, notes, e-books, etc.

Mark only one oval.

- Strongly agree
- Agree
- Neutral
- Disagree
- Strongly disagree

Attitude of medical students on LLMs in medical education

20. I am open to including LLMs as extra learning tools for medical studies *

Mark only one oval.

- Strongly agree
- Agree
- Neutral
- Disagree
- Strongly disagree

21. It would help by providing comprehensive medical information *

Mark only one oval.

- Strongly agree
- Agree
- Neutral
- Disagree
- Strongly disagree

22. Medical colleges should promote the use of LLMs in the teaching-learning process *

Mark only one oval.

- Strongly agree
- Agree
- Neutral
- Disagree
- Strongly disagree

23. LLMs could change how we learn and access medical knowledge *

Mark only one oval.

- Strongly agree
- Agree
- Neutral
- Disagree
- Strongly disagree

24. Relying too much on LLMs might not develop my clinical reasoning skills *

Mark only one oval.

- Strongly agree
- Agree
- Neutral
- Disagree
- Strongly disagree

25. There is a risk of learning the wrong concept; hence, I would not blindly believe it *

Mark only one oval.

- Strongly agree
- Agree
- Neutral
- Disagree
- Strongly disagree

Practice of medical students on large LLMs in medical education

26. I use LLMs to get clearer explanations of medical topics I'm learning *

Mark only one oval.

- Strongly agree
- Agree
- Neutral
- Disagree
- Strongly disagree

27. LLMs have shown me new resources and references for my medical studies *

Mark only one oval.

- Strongly agree
- Agree
- Neutral
- Disagree
- Strongly disagree

28. I use those only when I cannot get the information in a book *

Mark only one oval.

- Strongly agree
- Agree
- Neutral
- Disagree
- Strongly disagree

29. I use those only when I cannot get the information in Google or other search engine *

Mark only one oval.

- Strongly agree
- Agree
- Neutral
- Disagree
- Strongly disagree

30. I adapt my self-study based on insights I get from LLMs *

Mark only one oval.

- Strongly agree
- Agree
- Neutral
- Disagree
- Strongly disagree

31. Using LLMs has made me more confident in talking about medical subjects *

Mark only one oval.

- Strongly agree
- Agree
- Neutral
- Disagree
- Strongly disagree

This content is neither created nor endorsed by Google.

Google Forms
